# Dual adaptive strategies in *Candida glabrata* under tunicamycin stress: petite mutations and chromosome C aneuploidy drive transient drug resistance

**DOI:** 10.3389/fmicb.2025.1675175

**Published:** 2025-10-21

**Authors:** Yubo Dong, Chunhua Ma, Jing Wang, Shuai Bai, Chen Wang, Yi Xu

**Affiliations:** ^1^Department of Clinical Pharmacy, The 960th Hospital of PLA, Jinan, China; ^2^Pharmacy Intravenous Admixture Service, Department of Pharmacy, Zibo Zhoucun People's Hospital, Zibo, China

**Keywords:** *Candida glabrata*, tunicamycin, petite, transient resistance, fluconazole, aneuploidy

## Abstract

**Background:**

*Candida glabrata* is an opportunistic fungal pathogen known for its ability to rapidly develop resistance to antifungal agents. Tunicamycin (TUN), an inhibitor of N-linked glycosylation, induces Endoplasmic Reticulum (ER) stress, but the adaptive mechanisms enabling *C. glabrata* to survive TUN exposure remain poorly understood.

**Objective:**

This study aimed to identify and characterize the genetic and phenotypic adaptations that confer TUN resistance in *C. glabrata* and evaluate their stability in the absence of drug pressure.

**Methods:**

We exposed *C. glabrata* strain BG2 to sub-inhibitory (0.5 μg/mL) and inhibitory (1–8 μg/mL) TUN concentrations and isolated resistant mutants. Phenotypic characterization included growth assays, mitochondrial function tests (YPG medium), and fluconazole (FLC) susceptibility testing. Whole-genome sequencing assessed chromosomal alterations, and serial passaging in drug-free medium evaluated adaptation stability.

**Results:**

Under TUN stress, *C. glabrata* adopted two distinct resistance strategies: (1) mitochondrial dysfunction (petite formation), which conferred cross-resistance to FLC, and (2) aneuploidy, particularly disomy of chromosome C (ChrCx2), often accompanied by additional chromosomal gains in high-TUN conditions. However, both adaptations exhibited significant trade-offs: petite mutants retained irreversible respiratory deficiency but lost TUN and FLC resistance upon passaging, while aneuploid strains rapidly reverted to euploidy in non-selective conditions, abolishing TUN resistance.

**Conclusion:**

*C. glabrata* survives TUN stress through unstable genetic adaptations—petite formation and aneuploidy—that are rapidly selected against in drug-free environments. These findings highlight the evolutionary constraints of antifungal resistance mechanisms and suggest that intermittent therapy may help counteract resistance development.

## Introduction

The opportunistic fungal pathogen *Candida glabrata* has emerged as a major cause of invasive candidiasis (Katsipoulaki et al., 2024), exhibiting elevated resistance to common antifungals, particularly azoles (Beardsley et al., 2024). This species' remarkable ability to adapt to drug pressure stems from its genomic plasticity, including rapid acquisition of mutations and aneuploidies (Duggan and Usher, 2023). Understanding these adaptive mechanisms is crucial for developing effective antifungal strategies, particularly against drugs targeting essential cellular processes like protein glycosylation.

Tunicamycin (TUN), a nucleoside antibiotic that inhibits N-linked glycosylation, induces severe Endoplasmic Reticulum (ER) stress by disrupting proper protein folding (Wu et al., 2018). While not clinically used, TUN serves as an important tool for studying fungal stress responses and resistance mechanisms relevant to other antifungal agents (Yang et al., 2021a; Zheng et al., 2025b). Previous work in *Saccharomyces cerevisiae* has shown that ER stress can select for both mitochondrial dysfunction (petite mutants) and chromosomal aneuploidies (Beaupere et al., 2018). However, the adaptive strategies employed by *C. glabrata* under TUN pressure remain poorly characterized, despite this pathogen's clinical importance and unique stress response pathways.

Two particularly intriguing adaptation routes observed across fungal species include: (1) the formation of respiratory-deficient petite mutants, often associated with altered susceptibility to antifungal drugs (Brun et al., 2004, 2005; Cheng et al., 2007; Siscar-Lewin et al., 2021; Zheng et al., 2025a), and (2) the development of specific aneuploidies that provide transient resistance advantages (Selmecki et al., 2006; Polakova et al., 2009; Vande Zande et al., 2023). In *C. albicans*, for instance, chromosome 5 aneuploidy confers azole resistance but carries significant fitness costs (Selmecki et al., 2006). Whether *C. glabrata* employs similar strategies under ER stress, and how stable these adaptations might be, remains unknown.

This study aimed to systematically characterize *C. glabrata's* adaptive responses to TUN-induced stress across a range of drug concentrations. We specifically sought to: (i) identify the predominant resistance mechanisms emerging under both sub-inhibitory and inhibitory TUN concentrations, (ii) evaluate the stability of these adaptations in drug-free conditions, and (iii) assess potential cross-resistance implications for clinically relevant antifungals. Our findings reveal fundamental aspects of fungal evolutionary adaptation with potential implications for understanding and managing antifungal resistance.

## Materials and methods

### Strains and growth conditions

The *C. glabrata* reference strain BG2 served as the progenitor for this study. Stock cultures were preserved in 25% glycerol and stored at−80 °C. Cells were routinely cultured in Yeast Extract-Peptone-Dextrose (YPD) medium, which contains 1% (w/v) yeast extract, 2% (w/v) peptone, and 2% (w/v) D-glucose, at 30 °C using a shaking incubator set to 150-200 rpm. For YPG medium, the composition included 1% (w/v) yeast extract, 2% (w/v) peptone, and 3% (w/v) glycerol, with 2% (w/v) agar added for solid media. Drug solutions were prepared in dimethyl sulfoxide (DMSO) and stored at−20 °C.

### Growth curve

Cells were suspended in YPD broth. Cell densities were adjusted to 2.5 x 10^3^ cells/ml in YPD broth with or without TUN in 96 well plate. The plate was incubated at 30 °C. OD_595_ was monitored in a Tecan plate reader (Infinite F200 PRO, Tecan, Switzerland) at 15 min time intervals for 24 h. Data are represented as the mean ± SD of three biological replicates.

### Selection of TUN-resistant isolates adapted to sub-inhibitory concentrations

Cells were pre-cultured in YPD broth supplemented with a sub-inhibitory concentration of TUN (0.5 μg/mL) for 24 h. Cultures were then washed twice with sterile distilled water and serially diluted. Approximately 200 cells (from appropriate dilutions) were spread onto YPD agar plates. After incubation, 99 random colonies were isolated and screened for TUN resistance via spot assay. This procedure was performed in three independent biological replicates.

### Selection of TUN-resistant mutants using high-concentration screening

Cell suspensions were prepared in sterile distilled water and adjusted to a density of 1 × 107 cells/mL. Then, 100 μL of this suspension was spread evenly onto YPD agar plates supplemented with 8 μg/mL TUN. Plates were incubated at 30 °C for 5 days to select for resistant mutants. Following incubation, random colonies were isolated from TUN-containing plates for further analysis.

### Disk diffusion assay

Disk diffusion assays were performed according to the protocols outlined in our previous studies (Guo et al., 2024; Zheng et al., 2024a,b), following the CLSI M44-A2 guidelines for antifungal disk diffusion susceptibility testing (CLSI, 2009), with minor modifications. Briefly, strains were streaked from glycerol stocks onto YPD agar plates and incubated at 30 °C for 48 h. Colonies were then suspended in distilled water and adjusted to a concentration of 1 × 10^6^ cells/mL. A volume of 100 μL of this cell suspension was evenly spread across YPD plates. An empty paper disk (6 mm diameter and 0.7 mm thickness) was saturated with 5 μL of 40 mg/mL FLC and placed at the center of each plate. The plates were subsequently incubated at 30 °C and photographed after 48 h.

### Spot assay

Cells were suspended in distilled water and adjusted to a concentration of 1 × 10^7^ cells/mL. A volume of 3 μL of the cell suspension was spotted onto YPD or YPG plates. For testing susceptibility to TUN and NaCl, 3 μL of 10-fold serial dilutions were spotted on YPD plates containing 8 μg/mL TUN or 1.5 M NaCl. For tetrazolium dye reduction assay, YPD plate was supplemented with 0.04% tetrazolium. The plates were incubated at 30°C and photographed after 48 h.

### Broth microdilution assay

The Minimum Inhibitory Concentration (MIC) of FLC was determined following the Clinical and Laboratory Standards Institute (CLSI) broth microdilution method (CLSI, 2017) with slight modifications. Briefly, yeast cells from the mid-logarithmic growth phase were harvested, washed twice, and resuspended in sterile distilled water. The cell suspension was then diluted in YPD broth to a final density of 2.5 × 103 cells/mL, with FLC concentrations ranging from 0.125 to 128 μg/mL. Aliquots of 200 μL from each dilution were dispensed into 96-well microtiter plates. The plates were incubated statically at 30 °C for 48 h, after which the optical density at 600 nm (OD_600_) was measured using a microplate reader. All experiments were performed in triplicate, and growth control wells (YPD broth without FLC) were included for each strain.

### RNA extraction, synthesis of complementary DNA and quantitative real-time PCR

To analyze *PDR1* expression, test strains were cultured in YPD broth to the mid-logarithmic growth phase (OD_600_ = 1.0). For the TUN treatment experiment, cultures of the wild-type strain (BG2) were aliquoted into two flasks. One culture was supplemented with TUN to a final concentration of 8 μg/mL, while the other received an equivalent volume of vehicle as an untreated control. Both cultures were incubated for 3 h at 30 °C with shaking. For the comparative analysis between the petite mutant and BG2, both strains were grown separately to the mid-logarithmic phase without treatment. Cells from all conditions were harvested by centrifugation, and the pellets were immediately flash-frozen in dry ice and stored at−80 °C until RNA extraction.

Total RNA was isolated from the cell pellets using the YeaStar RNA Kit (Zymo Research) according to the manufacturer's instructions. RNA concentration and purity were determined spectrophotometrically (NanoDrop 2000C; Thermo Fisher Scientific) by measuring the absorbance ratios at 260/280 nm and 260/230 nm. RNA integrity was verified for selected samples by electrophoresis on 1% agarose gels.

Potential genomic DNA contamination was removed by treating approximately 1 μg of total RNA with DNase I (Thermo Fisher Scientific) at 37 °C for 30 mins. Reverse transcription (RT) was then performed using the High-Capacity cDNA Reverse Transcription Kit (Thermo Fisher Scientific) following the provided protocol.

The relative expression of PDR1 was quantified by qRT-PCR using the CFX96 Touch Real-Time PCR Detection System (Bio-Rad). The ACT1 gene was used as an endogenous control for normalization. Each qRT-PCR reaction was performed in triplicate. The comparative threshold cycle (2^∧^−ΔΔCT) method was employed to calculate the relative fold changes in gene expression (Schmittgen and Livak, 2008). Each reaction was performed in triplicate, and mean values of relative expression were determined for each gene.

### Whole-genome sequencing

DNA extraction, library construction and sequencing were performed as described previously (Yang et al., 2021b). Data was visualized using Ymap (Abbey et al., 2014). Raw fastq files were uploaded to YMAP (version 1.0) (http://lovelace.cs.umn.edu/Ymap/). Read depth was plotted as a function of chromosome position using the BG2 reference genome (https://www.ncbi.nlm.nih.gov/datasets/genome/GCA_014217725.1/).

### Daily passage of euploid petite isolates in YPD broth

Cryopreserved petite isolates were streaked aseptically onto YPD agar plates from −80 °C stocks. After 48h incubation at 30 °C, a single colony from each isolate was inoculated into 1 mL YPD broth. Cultures were incubated for 24h at 30 °C with shaking (200 rpm). For serial passaging, 1 μL of each culture was transferred daily into 1 mL fresh YPD broth. Following 10 passages, cells were harvested by centrifugation, washed twice with sterile distilled water, and resuspended. Suspensions were spread onto YPD agar plates. After incubation, one random colony per replicate was selected for downstream analysis.

## Results

### Sub-inhibitory tunicamycin pressure drives dual adaptation pathways in *Candida glabrata*: petite mutants and chromosome C aneuploidy

**Figure 1:**
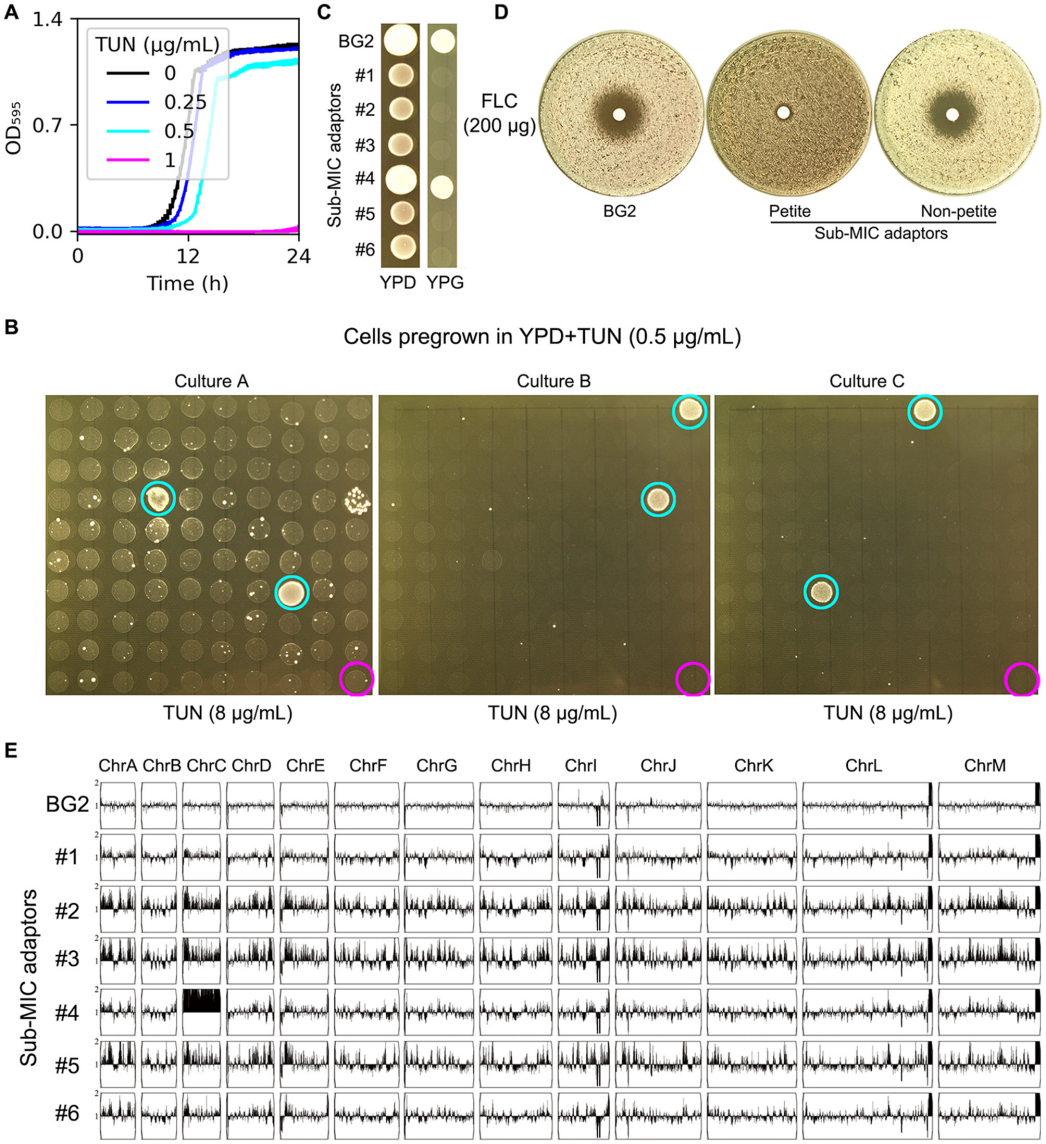
Characterization of TUN-resistant isolates selected by sub-inhibitory concentrations. **(A)** Growth kinetics of strain BG2 in YPD broth supplemented with TUN (0.25-1 μg/mL). Optical density was measured at 15-min intervals in a Tecan plate reader. Data represent means ± SD of three biological replicates. **(B)** TUN resistance screening of isolates pre-cultured in 0.5 μg/mL TUN. Ninety-nine random colonies from each of three independent cultures were assessed by spot assay. Cyan circles: TUN-resistant isolates; magenta circles: parental strain (BG2). **(C)** Mitochondrial function assessment. Cell suspensions (3 μL of 10^6^ cells/mL) of TUN-resistant isolates and parental strain were spotted on YPD (control) and YPG (glycerol as sole carbon source) plates. **(D)** Fluconazole susceptibility testing. Disk diffusion assays were performed with 200 μg fluconazole disks on TUN-resistant isolates and parental strain. **(E)** Genomic analysis of six TUN-resistant isolates. Karyotypes were visualized using YMAP following whole-genome sequencing.

We first determined the susceptibility of the parental *C. glabrata* strain BG2 to TUN by assessing growth in YPD broth supplemented with increasing TUN concentrations. While concentrations up to 0.5 μg/mL showed no significant growth inhibition (*P* > 0.05, Tukey's HSD test), 1 μg/mL TUN completely suppressed growth (Figure 1A).

To identify adaptive mechanisms under mild drug pressure, we exposed BG2 cells to a sub-inhibitory TUN concentration (0.5 μg/mL) for 24 h. From this population, we screened 100 colonies per biological replicate (*n* = 3) for resistance to high-dose TUN (8 μg/mL). Six resistant colonies (adaptors #1–6) emerged (Figure 1B, cyan circles), while parental BG2 remained sensitive (Figure 1B, magenta circles). Phenotypic characterization revealed two distinct resistance strategies: Five adaptors (#1–3, #5–6) exhibited a petite phenotype (YPG-negative, Figure 1C) and coincidentally acquired FLC resistance (no inhibition zone in disk assay, Figure 1D). The remaining adaptor (#4) maintained respiratory competence and harbored chromosome C disomy (ChrCx2), confirmed by whole-genome sequencing (Figure 1E).

These results demonstrate that sub-inhibitory TUN selects for either mitochondrial dysfunction (petite formation) or aneuploidy (ChrC disomy) in *C. glabrata*.

### Petite formation and aneuploidy persist as dominant adaptation strategies under high tunicamycin stress

**Figure 2:**
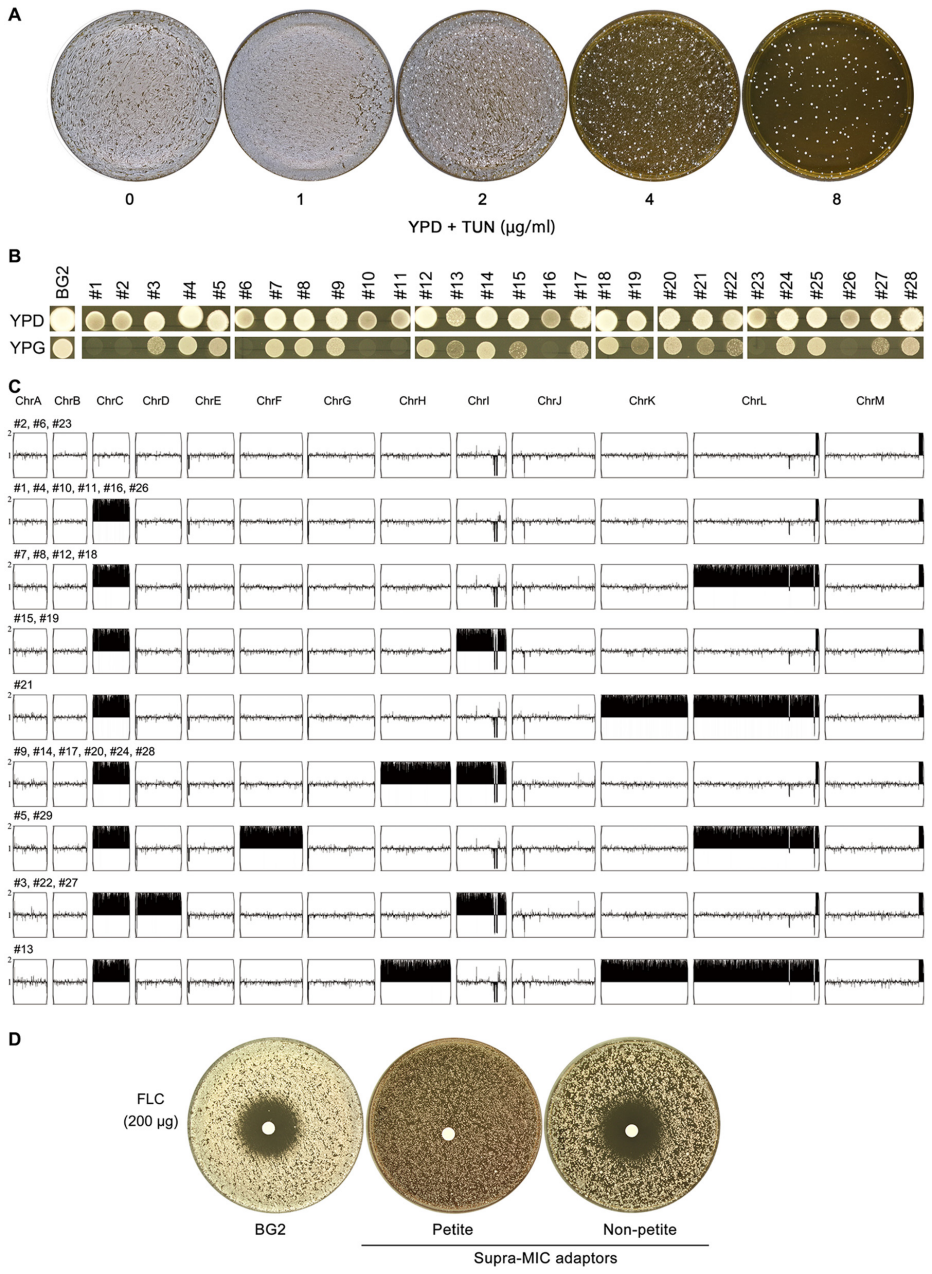
Characterization of TUN-resistant isolates selected by high-concentration screening **(A)** Colony formation of BG2 on YPD agar containing TUN (1–8 μg/mL). Plates were inoculated with 10^6^ cells and incubated at 30 °C for 3 days. **(B)** Mitochondrial function assessment of 28 TUN-resistant isolates. Growth was evaluated on YPG plates (glycerol sole carbon source) after incubation at 30 °C for 48h. **(C)** Karyotypic analysis of resistant isolates. Whole-genome sequencing was performed on all 28 isolates, with representative unique karyotypes visualized using Ymap. Numbers indicate isolates sharing each karyotype pattern. **(D)** Fluconazole susceptibility testing. Disk diffusion assays were performed with 200 μg fluconazole disks on TUN-resistant isolates and parental strain (BG2).

To isolate mutants under strong TUN pressure, we plated ~106 BG2 cells on YPD agar containing 1–8 μg/mL TUN. Resistant colonies emerged only at 8 μg/mL (Figure 2A). Among 30 randomly selected adaptors (excluding two with severe growth defects), all exhibited enhanced TUN resistance. Phenotypic analysis revealed: 8 petite mutants (YPG-negative, Figure 2B), including 3 euploid strains. Twenty five respiratory-competent adaptors, all aneuploid with ChrCx2 as the universal driver. These included: 6 single disomies (ChrCx2), 10 double disomies (ChrCx2+ChrDx2 or ChrCx2+ChrIx2), 9 complex aneuploidies (triple/quadruple disomies or other combinations) (Figure 2C). Notably, FLC resistance was exclusively linked to the petite phenotype, while non-petite adaptors (even with multiple disomies) remained FLC-sensitive (Figure 2D).

To definitively confirm the respiratory deficiency indicated by the growth assays on YPG, we directly assessed mitochondrial function using a tetrazolium dye reduction assay. This test provides a direct visual readout of respiratory competence, based on the reduction of a colorless tetrazolium salt to a red formazan precipitate by active mitochondrial electron transport chains (Berridge et al., 2005). We tested a total of 13 adaptors: five from sub-MIC and eight from supra-MIC conditions. The results demonstrated a perfect correlation: all mutants unable to grow on glycerol also failed to produce the formazan precipitate (Supplementary Figure 1). This unequivocally confirms that the growth defect is due to a loss of respiratory function, establishing true petite phenotype in these adaptors.

### Phenotypic instability of euploid petite adaptors

**Figure 3:**
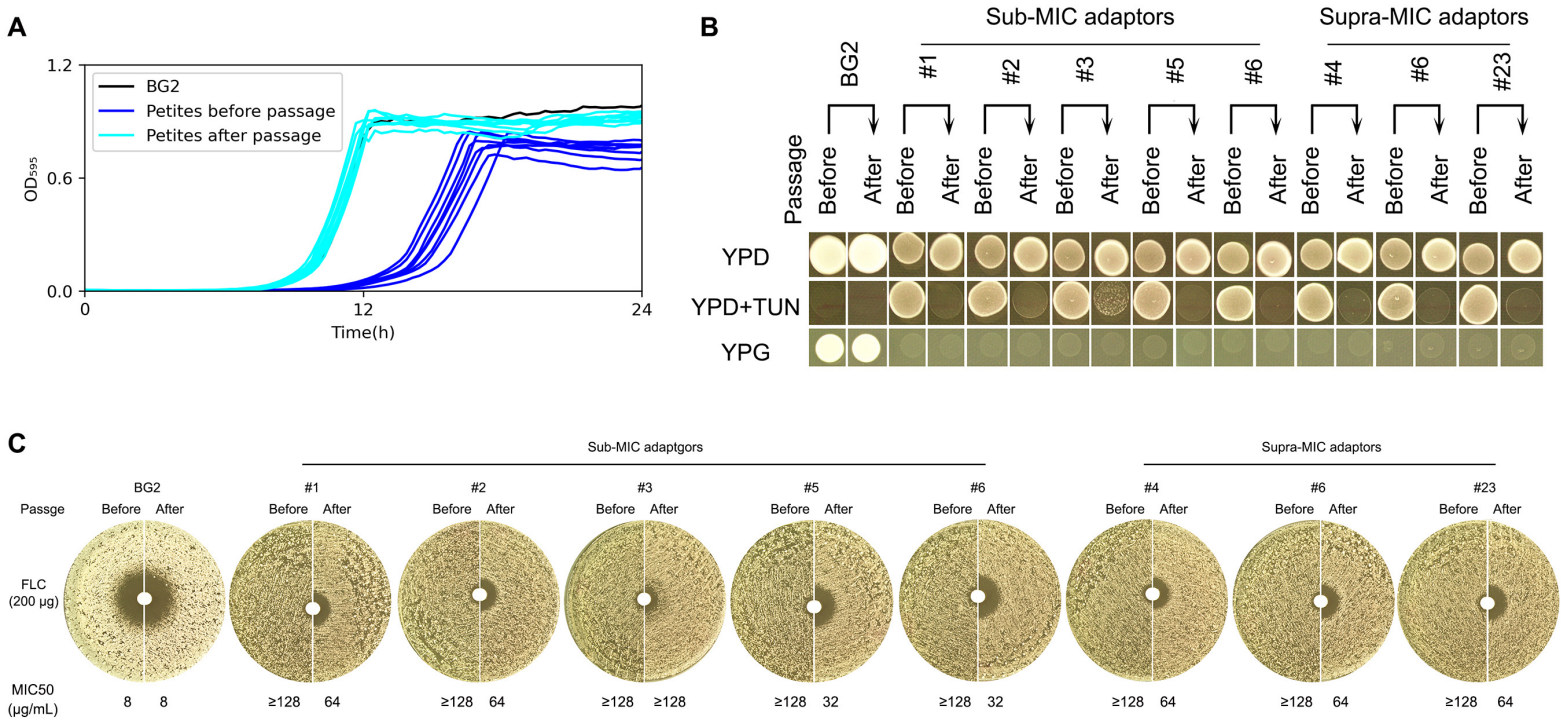
Phenotypic stability assessment of euploid TUN-resistant isolates during serial passaging Euploid TUN-resistant isolates selected under both sub- and supra-MIC TUN concentrations were serially passaged in YPD broth. **(A)** Growth fitness comparison. Growth curves in YPD broth were measured for pre-passage and post-passage isolates using a plate reader (15-min intervals, 30 °C). **(B)** TUN resistance and mitochondrial function. Pre-passage vs. post-passage isolates were compared for TUN resistance (spot assay) and mitochondrial deficiency (YPG growth). **(C)** Fluconazole susceptibility. Disk diffusion assays (200 μg fluconazole disks) evaluated resistance changes after passaging.

To evaluate the long-term stability of drug-resistant adaptations, we subjected eight euploid petite mutants (five isolated under sub-MIC and three under supra-MIC TUN conditions) to ten serial passages in non-selective YPD medium. Following this experimental evolution, four key phenotypic changes emerged: First, all evolved isolates demonstrated significantly improved growth kinetics in rich medium (*p* < 0.001, Tukey's HSD test; Figure 3A), consistent with the acquisition of compensatory mutations that ameliorate the fitness cost associated with petite formation. Second, despite this improved growth, every passaged isolate completely lost its original TUN resistance (Figure 3B), strongly suggesting that the maintenance of TUN resistance mechanisms imposes a metabolic burden that becomes unfavorable in the absence of drug selection pressure. Third, in contrast to the transient nature of drug resistance, the respiratory-deficient phenotype remained completely stable, with all mutants maintaining their inability to grow on YPG medium (Figure 3B), indicating that the underlying mitochondrial dysfunction results from either irreversible mtDNA mutations or stable nuclear mutations affecting oxidative phosphorylation. Finally, we observed a partial attenuation of FLC resistance in passaged isolates, evidenced by significantly larger inhibition zones in disk diffusion assays, and reduced MIC_50_ values compared to their ancestors (Figure 3C), implying that the genetic determinants of FLC resistance may be either pleiotropically linked to or co-dependent upon the same unstable elements that confer TUN resistance.

### Genomic and phenotypic instability of aneuploid non-petite adaptors

**Figure 4:**
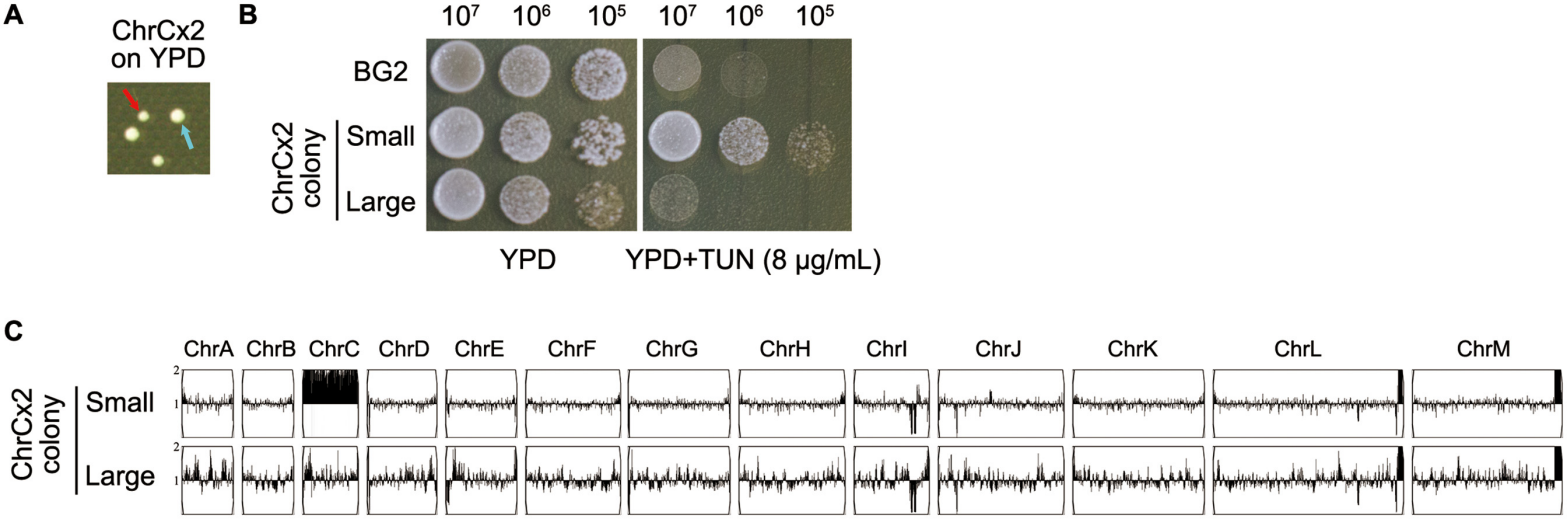
Phenotypic and genomic instability analysis of ChrCx2 mutant isolate **(A)** Colony polymorphism on YPD agar. Cells of ChrCx2 were plated and incubated at 30 °C for 48h. Red arrow: small colony variant; cyan arrow: large colony variant. **(B)** TUN resistance profiling. Small and large isolates were compared to parental strain (BG2) using spot assays on YPD ± 8 μg/mL TUN. **(C)** Karyotypic stability assessment. Whole-genome sequencing of small and large colonies was performed, with karyotypes visualized using Ymap against the BG2 reference.

Phenotypic and genotypic analyses of aneuploid *C. glabrata* strains harboring ChrCx2 demonstrated marked genomic instability following short-term growth on non-selective media, even without extended passaging. Plating experiments on YPD medium revealed a striking colony size polymorphism, with two distinct morphotypes emerging: small colonies (Figure 4A, red arrow) maintained the ChrCx2 aneuploidy and retained TUN resistance, while large colonies (Figure 4A, cyan arrow) uniformly reverted to euploidy and showed restored drug sensitivity comparable to wild-type BG2 (Figures 4B, C). Whole-genome sequencing confirmed this genotype-phenotype correlation, demonstrating that the fitness advantage of euploid revertants in drug-free conditions drives strong selection against the aneuploid state. These findings provide compelling evidence that while ChrC disomy serves as an effective short-term adaptation to TUN stress, the associated genomic imbalance imposes significant fitness costs that promote rapid reversion to euploidy when selective pressure is removed. This dynamic equilibrium between adaptive aneuploidy and restorative euploidy highlights the fundamental trade-off between rapid stress adaptation and long-term genomic stability in fungal pathogens.

## Discussion

Our study reveals two distinct but evolutionarily unstable adaptive strategies employed by *C. glabrata* to overcome TUN stress: mitochondrial dysfunction (petite formation) and Chromosome C disomy formation. These findings significantly advance our understanding of fungal stress adaptation by demonstrating how sublethal drug pressure drives divergent evolutionary trajectories with important implications for antifungal resistance and pathogen evolution.

The emergence of petite mutants under both sub-inhibitory (0.5 μg/mL) and lethal (8 μg/mL) TUN concentrations points to mitochondrial dysfunction as a primary adaptive strategy to escape the effects of TUN in *C. glabrata*. In line with this concept, it has been independently reported that deliberate induction of mitochondrial dysfunction via deletion of *MIP1* (encoding mitochondrial DNA polymerase) results in TUN resistance in this yeast (Siscar-Lewin et al., 2021). Thus, both pharmacological and genetic evidence point to the same adaptive endpoint. This phenomenon is not without precedent, as it parallels observations in *S. cerevisiae*, where ER stress similarly triggers petite formation (Beaupere et al., 2018).

The consistent association between petite formation and FLC cross-resistance (Figures 1D, 2D) suggests a shared adaptive mechanism, potentially involving alterations in sterol biosynthesis and/or efflux pump regulation. In support of this hypothesis, we found that expression of *PDR1*—which encodes a zinc-finger transcription factor that activates FLC efflux genes—was significantly upregulated in the petite mutant compared to the wild-type BG2 (fold change = 3.6 ± 0.7; *p* < 0.001, Student's *t*-test). Concurrently, the increased susceptibility of petite mutants to the membrane stressor NaCl (Supplementary Figure 1) further suggests possible defects in membrane integrity, consistent with the idea that altered sterol biosynthesis may contribute to their fitness profile under stress.

Notably, while the respiratory-deficient phenotype remained stable during serial passage, the rapid loss of both TUN and attenuated FLC resistance in non-selective conditions (Figure 3) implies that these resistance mechanisms carry substantial fitness costs. This instability may explain the transient nature of drug resistance in clinical isolates following antifungal withdrawal.

Chromosome C disomy emerged as the second major adaptive strategy, exhibiting several remarkable features. First, ChrCx2 was universally present in all respiratory-competent adaptors (Figure 2C), suggesting specific genes on this chromosome facilitate TUN resistance. Potential candidates include the putative oligosaccharyltransferase complex components or other ER stress response elements located on chromosome C. Second, the frequent occurrence of additional disomies (ChrD, ChrI, etc.) in high-TUN adaptors suggests a cumulative gene dosage effect may be required for resistance under extreme stress. This parallels findings in *C. albicans*, where progressive aneuploidy enhances drug resistance (Wang et al., 2025).

The instability of both adaptive strategies reveals fundamental evolutionary constraints. For petites, while mitochondrial mutations are irreversible (Figure 3B), the associated drug resistance is genetically unstable. For aneuploids, the rapid reversion to euploidy in drug-free conditions (Figure 4) confirms the substantial fitness cost of chromosomal imbalances. This mirrors observations in *Saccharomyces cerevisiae*, where aneuploidy provides transient adaptive benefits but is ultimately selected against in stable environments (Yona et al., 2012).

These findings have important clinical implications. First, the dual adaptation pathways suggest *C. glabrata* possesses remarkable plasticity in overcoming ER stress, which may contribute to its success as an opportunistic pathogen. Second, the instability of these adaptations implies that resistant clinical isolates may revert to susceptibility when drug pressure is removed, supporting intermittent antifungal strategies. Finally, the consistent linkage between petite formation and FLC cross-resistance (Figures 1D, 2D) suggests that preventing this adaptive mitochondrial dysfunction—rather than inducing it—could be a novel strategy for combination therapies by stabilizing mitochondrial function to block a key pathway to resistance.

Future studies should investigate: (1) The specific ChrC genes responsible for TUN resistance, (2) The molecular basis of FLC cross-resistance in petites, and (3) Whether clinical isolates show similar adaptation patterns. Understanding these mechanisms will be crucial for developing strategies to counteract fungal adaptation and prevent resistance emergence.

In conclusion, our work demonstrates that *C. glabrata* employs two distinct but evolutionarily constrained strategies to overcome TUN stress, highlighting the complex trade-offs between rapid adaptation and long-term fitness that shape fungal evolution under drug pressure.

## Data Availability

The sequencing data have been deposited in the ArrayExpress database at EMBL-EBI (www.ebi.ac.uk/arrayexpress) under accession number E-MTAB421 12427, E-MTAB-12423 and E-MTAB-12484.
