# Supplementary figures and images for: Dual adaptive strategies in Candida glabrata under tunicamycin stress: petite mutations and chromosome C aneuploidy drive transient drug resistance
Source: Front Microbiol. 2025 Oct 21;16:1675175. doi: 10.3389/fmicb.2025.1675175 (PMC12583059; doi:10.3389/fmicb.2025.1675175)

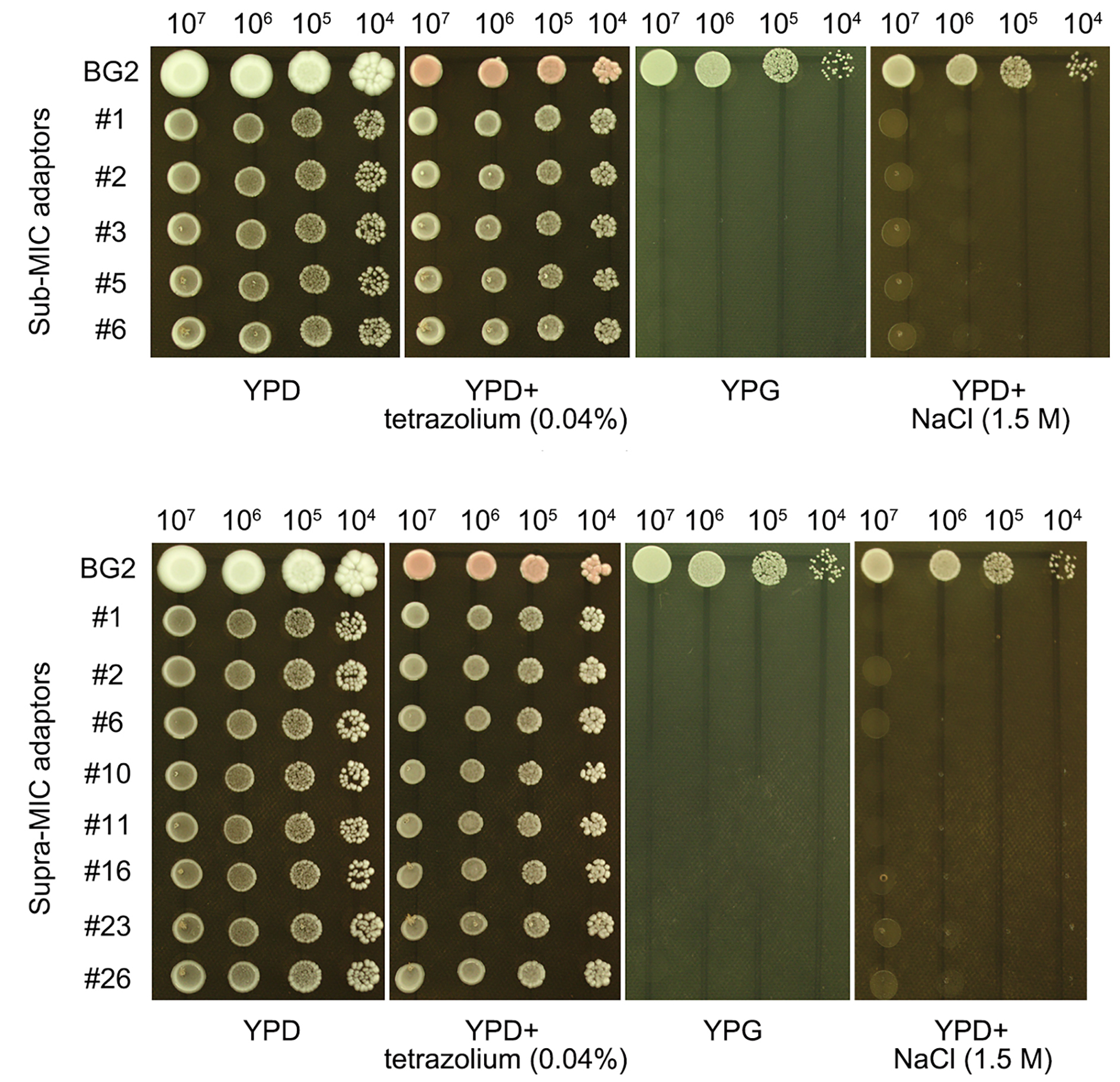

Supplement: Supplementary file 1 [file Image_1.jpeg]
